# Letters to the Editor

**Published:** 1909-06

**Authors:** 


### Letters to the Editor.

COMMENDATION FROM ROCHESTER, N. Y., TO CITY OF NEW MEXICO. REACH OF THE "RED BACK."

Mexico, D. F., April 28, 1909.

*The Texas Medical Journal, Austin, Texas.*

My Dear Doctor: Through pure cussed neglect I am just sending you eight pesos in Mexican money, which I suppose will be $4.00 of God's currency. This is to pay for the "Red Back" for a while, at least. I would not like to see the Journal miss a single number, for it would give me a sort of constipated feeling, or a "busted tire" sensation.

Why don't you come down to Mexico some time, where you get cool in the summer and warm in the winter, and plenty of everything good all the year round.

Wishing you and yours many years of happy life and of doing good in your special field, I am,

Faithfully yours,

CHAS M. HARRISON.

---

GREENVILLE, TEXAS, May 1, 1909.

*Dr. F. E. Daniel, Austin, Texas.*

MY DEAR DOCTOR: Please find enclosed postoffice order for $1.00 subscription to "Red Back." Thanking you for your kind indulgence, and admiring you for the way you "speak out in meeting," I shall continue to take the "Red Back," if you will let me.

Very truly,

T. J. MILNER.

---

AURORA, MISSOURI, April 24, 1909.

*F. E. Daniel, M. D., Austin, Texas.*

DEAR DOCTOR: Enclosed find check for $1.00. Please send TEXAS MEDICAL JOURNAL one year, commencing with the May number. You are doing good work. Your efforts are appreciated.

Yours truly,

F. S. STEVENSON.

---

FORT WORTH, TEXAS, May 3, 1909.

*Dr. F. E. Daniel, Austin, Texas.*

DEAR DOCTOR: Inclosed find check for $3.00, to pay for the only good journal that we have to day. *Let the hot shots continue.*

Your truly,

W. B. MACKEY, M. D.

---

623 PORTLAND AVENUE, ROCHESTER, N. Y., April 27, 1909.

*F. E. Daniel, M. D., Editor Texas Medical Journal, Austin, Texas.*

DEAR DOCTOR: I received a copy of the famous "Red Back" just a few days before leaving this city for New York and other places, in which I was gathering material. You may believe that I read with great interest the fearless iconoclastic article of Dr.

Lydston. The truth is mighty and will prevail, is an old saying. But when one considers how little interest, how generally apathy is displayed and shown by the American people, both professional and lay, whenever a contest is to be waged between right and wrong, between principle and policy, the deductions and predictions drawn so well by you in your essay on the American people,* return to one's recollection with ominous, almost startling application and force. It can not be denied that the silent secret forces of national decay and disintegration have begun to do their deadly work. And for that very reason it is so necessary for the medical profession and for the clergy to open their eyes and grapple with the problems—those vital passing problems of physical and spiritual, individual and family, health and integrity, the further avoidance or neglect of which must inevitably hasten the work of the hidden causes already so alarmingly in evidence, especially in the East.

For this reason, I wrote the answer which you received to your first letter commending my previous communication. And, if you are able to comply with my request, I shall be pleased to have you print it in one of your future issues. I ask this because I wish first of all to pay a well-deserved tribute to the four physicians whom I have named and whom I honor for their self-sacrificing professional work. Then there is the second reason mentioned above, that of urging the profession to take more interest in the vital subject of individual and family integrity. I am not surprised to learn, as I have recently, that the need of this work is being felt no less in Europe than in America. Happily, however, Europe is not hampered or restrained by that false, irrational aversion so much in evidence in this supposed land of freedom. Only one who has specialized in this comparatively unpopular and untrodden field as I have, and has gathered his material not only from essays, lectures and works, but also from the frank expressions of adults, both single and married, can realize and know how closely connected these subjects are with almost every sociological question which is now so alarmingly in evidence in our family, civil and national life.

Wishing you continued and well-deserved success in your unselfish sturdy work as editor of the "Red Back," I am,

Very sincerely,

E. C. MARGRANDER.

---

*Elements of Decay in American Civilization by F. E. Daniel, M. D., Texas State Journal of Medicine, December, 1908.

